# Medical Representatives on Insurance Committees

**Published:** 1914-07-11

**Authors:** 


### THE PROFESSION OF MEDICINE.

### Medical Representatives on Insurance Committees.

In a circular distinguished as 46/I.C. the English Insurance Commissioners have issued regulations concerning the manner in which two representatives of the medical profession in each Insurance area shall be elected to serve on the Insurance Committee of that district. The term of office of those now serving expires on July 15. Nominations, to be effective, must have reached the Commissioners not later than noon on June 20, and any practitioner may be nominated who is entitled to a vote in the area served by that Committee. The register of those entitled to vote has been taken from the *Medical Register* corrected to June 1; but any qualified man who for any reason had a right to vote and was not placed on the voting register could obtain admission to it by submitting a claim for that purpose.

Nominations had to be signed by not less than five electors and also by the candidate as evidence that nomination was accepted. If the nominations in any district have not exceeded two, those candidates were declared duly elected on or after June 23. Otherwise the electors will have to ballot for their representatives. The probable abstention of non-panel medical men from these elections both as candidates and voters will doubtless result in the election of exclusively panel practitioners, a contingency which we cannot regard as desirable in the interests of the Committees, the insured persons, or the profession of medicine.

